# Hybrid Multi-Scale Neural Network with Attention-Based Fusion for Fruit Crop Disease Identification

**DOI:** 10.3390/jimaging11120440

**Published:** 2025-12-10

**Authors:** Shakhmaran Seilov, Akniyet Nurzhaubayev, Marat Baideldinov, Bibinur Zhursinbek, Medet Ashimgaliyev, Ainur Zhumadillayeva

**Affiliations:** 1 Faculty of Information Technologies, L.N. Gumilyov Eurasian National University, Astana 010000, Kazakhstan; seilov_shzh@enu.kz (S.S.); baideldinov_mu@enu.kz (M.B.); zhursinbek_bsh@enu.kz (B.Z.); ashimgaliyev_mzh_2@enu.kz (M.A.); 2 “Kazakh Academy of Infocommunications” Public Fund, Astana 010000, Kazakhstan; anurzhaubayev@kai.kz

**Keywords:** plant disease detection, deep learning in agriculture, leaf image classification, CNN-based diagnosis, feature fusion, multiscale image analysis, agricultural computer vision, early disease recognition, image-based crop health monitoring

## Abstract

Unobserved fruit crop illnesses are a major threat to agricultural productivity worldwide and frequently cause farmers to suffer large financial losses. Manual field inspection-based disease detection techniques are time-consuming, unreliable, and unsuitable for extensive monitoring. Deep learning approaches, in particular convolutional neural networks, have shown promise for automated plant disease identification, although they still face significant obstacles. These include poor generalization across complicated visual backdrops, limited resilience to different illness sizes, and high processing needs that make deployment on resource-constrained edge devices difficult. We suggest a Hybrid Multi-Scale Neural Network (HMCT-AF with GSAF) architecture for precise and effective fruit crop disease identification in order to overcome these drawbacks. In order to extract long-range dependencies, HMCT-AF with GSAF combines a Vision Transformer-based structural branch with multi-scale convolutional branches to capture both high-level contextual patterns and fine-grained local information. These disparate features are adaptively combined using a novel HMCT-AF with a GSAF module, which enhances model interpretability and classification performance. We conduct evaluations on both PlantVillage (controlled environment) and CLD (real-world in-field conditions), observing consistent performance gains that indicate strong resilience to natural lighting variations and background complexity. With an accuracy of up to 93.79%, HMCT-AF with GSAF outperforms vanilla Transformer models, EfficientNet, and traditional CNNs. These findings demonstrate how well the model captures scale-variant disease symptoms and how it may be used in real-time agricultural applications using hardware that is compatible with the edge. According to our research, HMCT-AF with GSAF presents a viable basis for intelligent, scalable plant disease monitoring systems in contemporary precision farming.

## 1. Introduction

Global food security depends heavily on agriculture, which is still an essential economic sector, especially for many developing countries [1,2]. However, fruit crop diseases continuously endanger the production of agriculture, resulting in significant yield losses and economic challenges. These diseases are caused by a variety of biological agents, such as bacteria, viruses, and fungi, in addition to adverse environmental factors, such as harsh climate and unhealthy soil [3,4]. Regretfully, farmers are frequently short of the knowledge and resources necessary for early detection of these diseases, particularly those who operate in remote or resource-constrained areas. This results in delayed interventions and increasing losses [4]

Fruit crop diseases must be identified early and accurately in order to prevent significant damage, maintain production, and reduce dependence on expensive and ecologically harmful treatments [5]. In agriculture, visual inspection by qualified professionals has historically played a major role in disease diagnosis. But these traditional approaches are time-consuming, expensive, subjective, and difficult to scale, especially in distant or impoverished areas where the availability of experts is low [6]. Inconsistencies and mistakes during disease identification are further compounded by the human constraints of subjectivity, weariness, and differing levels of competence.

Researchers are increasingly looking to automated solutions that use computer vision and artificial intelligence to address these issues. Since the majority of fruit crop illnesses show up visually on leaves and fruits, digital imaging has been very useful in recent years. In general, these automated systems have progressed from traditional machine learning methods that depend on attributes that are manually created to advanced deep learning methods, particularly convolutional neural networks (CNNs) [7].

Traditional machine learning approaches usually combine classifiers like Support Vector Machines (SVM) or Random Forests with feature extraction algorithms like Scale-Invariant Feature Transform (SIFT), Histogram of Oriented Gradients (HOG), or Local Binary Patterns (LBP). These techniques rely mostly on manually designed features, which restricts their capacity to adapt to changing settings like different lighting, scales, and illness symptoms, even when they achieve adequate accuracy [8,9,10].

By automating feature extraction straight from raw photos, deep learning—in particular, CNN-based models like ResNet, DenseNet, and EfficientNet—has greatly improved plant disease identification by improving generalization capabilities. However, CNNs confront a number of significant obstacles in real-world agricultural applications, even though they exhibit remarkable accuracy in controlled environments. Class imbalance in training datasets, vulnerability to performance degradation in complex field backgrounds, difficulty detecting early-stage diseases with subtle visual symptoms, and high computational resource requirements that impede deployment on edge devices frequently used in agricultural environments represent a few of the main problems [4,6,11].

More sophisticated neural network architectures that can efficiently capture disease symptoms at different dimensions and situations are required to overcome these constraints. Multi-scale architectures greatly improve detection accuracy across a range of symptom presentations by allowing models to examine both fine-grained local information and more general contextual patterns at the same time. Furthermore, new developments in Vision Transformers (ViTs) show promise in capturing global structural information and long-range relationships, which are features that conventional CNNs frequently ignore [12,13].

This paper presents a Hybrid Multi-Scale Neural Network (HMCT-AF with GSAF) architecture designed especially for fruit crop disease identification, driven by these advancements. Multiple convolutional branches specifically built for extracting disease features at different scales—from minor local symptoms to more significant, bigger disease manifestations—are integrated in our suggested method. In order to capture complex spatial relationships and global patterns throughout the entire image, we also include a structural-pattern branch based on the Vision Transformer architecture. Our method relies on a new attention-based feature fusion module that enhances the interpretability and robustness of the model by adaptively combining Transformer-derived features with multi-scale CNN features [14,15].

The study described here covers important issues in fruit crop disease detection, such as class imbalance, early disease identification, and variations in disease symptom presentation. We demonstrate the effectiveness of our approach across a range of fruit crop species and imaging settings by testing our HMCT-AF with GSAF on well-known benchmarks such as the PlantVillage and Cassava Leaf Disease datasets [12,16,17].

The format of this document is as follows: In Section 2, relevant work on automated fruit crop disease detection is reviewed; in Section 3, our proposed HMCT-AF with GSAF architecture is presented in detail; in Section 4, experimental setups and datasets are described; in Section 5, experimental results and comparative analysis are discussed; and in Section 6, the paper is concluded with insights and future research directions.

## 2. Literature Review

### 2.1. Traditional Machine Learning Approaches for Plant Disease Classification

Traditional classifiers and hand-crafted characteristics were key components of early attempts to identify plant diseases. Using a variety of image processing approaches, researchers manually retrieved pertinent information from leaf photos [18,19,20]. Researchers utilized traditional machine learning algorithms to categorize the leaves as either “healthy” or “diseased”

- Texture features (e.g., Local Binary Patterns, LBP) [21,22].
- Color features (e.g., color histograms) [18,22].
- Shape features (e.g., shape descriptors) [18,23]

These handcrafted features were fed into classifiers such as Support Vector Machines (SVM), decision trees, k-nearest neighbors (k-NN), and naive Bayes models [18,24].

Early systems for crop disease classification relied on statistical analysis of color and shape features, demonstrating reasonable accuracy for a limited number of disease types [20,21]. Some approaches combined color and texture descriptors with traditional classifiers like support vector machines to extend applicability across different crops. While these methods showed promise, their performance heavily depended on the quality of hand-crafted features [22,23,25]. They also struggled with complex backgrounds, varying lighting conditions, and overlapping visual symptoms. In addition, scalability was limited, as adding new disease categories often required retraining the model or redesigning the feature extraction process.

### 2.2. Deep Learning Methods for Plant Disease Classification

Plant disease diagnosis using images has changed with the introduction of deep learning, specifically convolutional neural networks (CNNs). CNNs do not require manual feature engineering because they automatically extract hierarchical features from unprocessed image data. The success of CNNs in general-purpose picture classification tasks (like ImageNet) encouraged academics to use them on datasets related to plant diseases [26,27,28,29,30].

In one of the first and most significant studies, it was used the PlantVillage dataset to train the AlexNet and GoogLeNet architectures, achieving an accuracy of over 99% under carefully monitored circumstances [31]. This illustrated the potential of classifying plant diseases using deep CNNs. Since then, plant leaf images have been used to test a variety of CNN architectures, including VGGNet, ResNet, DenseNet, and Inception, with promising outcomes. Custom-designed networks have also been developed to identify multiple disease categories, further highlighting the versatility of CNN-based approaches in this domain [32,33,34,35,36,37,38].

However, there are certain disadvantages to deep learning models. First off, field images often have noise, complex backdrops, fluctuating lighting, or numerous overlapping leaves, whereas most models work best on clear, lab-quality images. In real-world applications, these elements may drastically lower model accuracy. Second, deep network training requires huge datasets, strong GPUs, and a significant amount of time—all of which may not be possible in agricultural environments with restricted resources [39,40,41,42]. A further concern is overfitting, where models may learn patterns from training data, particularly if the dataset is imbalanced or restricted to particular crops or geographical areas

### 2.3. Multiscale and Multilevel Feature Extraction Approaches

By enhancing CNN architectures with multiscale and multilevel feature extraction, several works have tried to address the mentioned problems. While multilevel feature fusion allows the model to maintain both fine-grained features and high-level semantic information, multiscale approaches help in the capturing of lesions and disease symptoms at different sizes and shapes [33,43,44,45]. These enhancements have demonstrated the ability to improve robustness to intra-class variability and enable the early detection of disease.

For instance, CNN models now include atrous (dilated) convolutions and spatial pyramid pooling (SPP) layers to better identify small lesion regions that could otherwise go unnoticed. These techniques make the model more sensitive to tiny but crucial areas in the image by allowing the network to utilize wider receptive fields without significantly increasing the number of parameters [36,46,47]. This is particularly helpful in the context of plant disease when symptoms are minimal or only partially developed [48,49].

Furthermore, research has been performed on hybrid models that utilize feature-fusion strategies or combine multiple convolutional feature extractors. These models aim to enhance generalization and leverage the advantages of various network architectures. Some frameworks, for instance, use combination, addition, or attention techniques to combine features that have been extracted in parallel from various convolutional layers or stages. Others maintain the flow of features through deeper networks and address vanishing-gradient problems by introducing residual or dense connections across layers [34,50,51].

### 2.4. Data Augmentation and Class Imbalance Solutions

Enhancing learning through data augmentation and improved loss function design has been another crucial area. By producing altered copies of images (using transformations like flipping, rotating, scaling, or color shifting), data augmentation broadens the scope of constrained datasets. This method minimizes overfitting and enhances a model’s capacity for generalization [42]. For plant disease datasets, where it is challenging and expensive to gather a high number of annotated images for each class, augmentation is particularly important.

Modified loss functions have also been used by researchers to resolve class imbalance, which occurs when some disease classes are underrepresented. Methods like label smoothing, weighted cross-entropy, and focal loss modify the training objective to take unequal class distributions into consideration. Focal loss, for instance, drives the model to concentrate on more difficult, incorrectly categorized cases while down-weighting well-classified examples [40,41]. These methods are especially helpful when a disease is rare, but its precise identification is crucial.

### 2.5. Other Relevant Techniques

A number of other methods have been investigated to enhance plant disease classification in addition to typical CNN architectures. The model’s focus can be directed to the leaf image’s most informative areas (such as the lesion regions) by using attention mechanisms [33]. The ability of capsule networks to maintain spatial correlations between features has been studied; this could help capture complex patterns of disease symptoms. Additionally, transfer learning has shown great potential [46]. Researchers have been able to obtain high accuracy with a relatively minimal quantity of domain-specific training data by developing a model that was pretrained on a big dataset, such as ImageNet, and then fine-tuning it on data related to plant diseases [30,52,53].

The use of ensemble learning techniques has also shown potential. An ensemble combines the predictions of several models to produce a more reliable prediction as a total [33,36]. A voting scheme or averaging the outputs of multiple classifiers, for instance, can yield more reliable and accurate results under various test scenarios than any one model by itself [50,51].

### 2.6. Comparative Performance of Existing Methods

Deeper and more complicated models typically perform better, as long as there is enough training data, according to studies that examine various CNN architectures on benchmark datasets. Modern networks like DenseNet and ResNet, for instance, consistently perform better than smaller models like AlexNet or LeNet, particularly when it comes to identifying minor visual changes between illness classes. Nevertheless, these performance improvements frequently result in noticeably greater computing costs [34,35,45].

Consequently, high-performance yet lightweight architectures that are appropriate for field or mobile deployment are becoming more and more popular. To compress huge models while preserving the majority of their accuracy, methods including knowledge distillation, quantization, and model pruning are utilized. These initiatives support the broader objective of offering low-cost, low-power solutions to farmers and agricultural laborers in remote or underdeveloped regions [49,50,54].

### 2.7. Summary of Literature Review

In summary, the combination of deep learning and computer vision methods has significantly improved image-based plant disease identification. Although they offered a valuable starting point, traditional machine learning techniques have mostly been replaced by data-driven deep learning methods (especially CNNs). It is still difficult to continuously achieve good performance under real-world field settings, though. This gap is being filled by ongoing research using enhancements in data processing, feature extraction, and model development. To improve model robustness and generalization, for instance, multiscale/multilevel feature fusion, better loss functions for imbalance, and efficient augmentation techniques are being used.

As the area develops, more attention is being paid to models that are accurate, reliable, understandable, and useful for actual agricultural applications. Combining high diagnostic accuracy, computational efficiency, and environmental, disease, and agricultural adaptation is a major challenge. Translating the achievements of AI-based plant disease detection from the lab to the field will depend on striking this equilibrium [33,41,50].

## 3. Materials and Methods

### 3.1. Image Preprocessing and Augmentation

**Figure 1:**
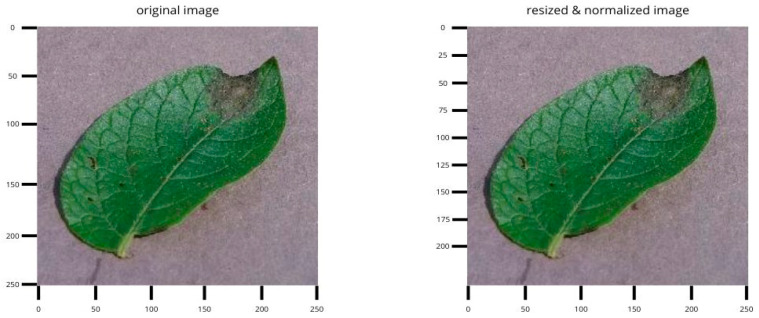
Image resizing and normalization applied to a sample image from the PlantVillage benchmark dataset.

Before uploading the images to the CNN model, we applied a number of preprocessing steps to ensure that they meet the input requirements of the model. The size of each image has been resized to 224 × 224 pixels to ensure consistency across the entire dataset. We also normalized the pixel values by dividing them by 255.0, which helps to standardize the data and promote more stable and efficient learning [29,30]. As shown in Figure 1, the preprocessing process includes resizing and normalization, shown next to the original image for comparison.

**Figure 2:**
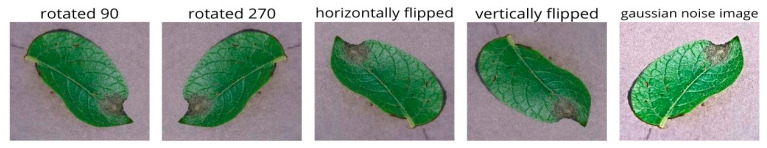
Image samples created using the PlantVillage benchmark image sample.

The effectiveness of models based on convolutional neural networks (CNNs) largely depends on the availability of a sufficiently large set of training samples. In the absence of sufficient data, there is a risk of overfitting—when the model learns patterns that are too specific for the training set and are difficult to generalize. To solve this problem, we expanded the dataset using three geometric data augmentation methods: rotation, flip, and noise addition [42]. Although these methods do not preserve labels in the strict sense, they help diversify the training set [44,47,49]. Several examples of augmented images are shown in Figure 2 [31].

### 3.2. Benchmark Acquisition

**Figure 3:**
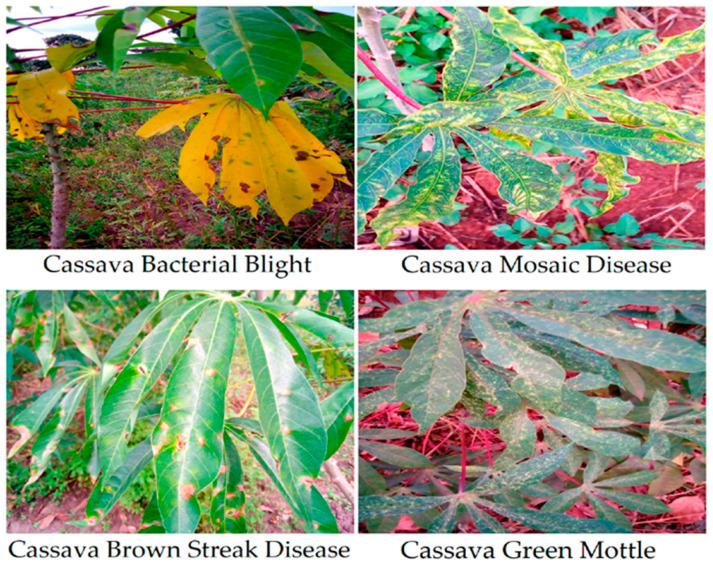
Image samples of Cassava Lead Disease dataset.

To evaluate the performance of the proposed model, we used 3 publicly available reference datasets: the Cassava Leaf Disease Dataset (CLD) and PlantVillage. The examples highlight variability in lighting, background, and symptom scale that motivates our multi-branch design. In this study, the CLD dataset was primarily used to evaluate the effectiveness of the model. It consists of 21,397 labeled cassava leaf images divided into five separate classes [53]: (i) Healthy Cassava Leaves (HCL), (ii) Cassava Bacterial Blotch (CBB), (iii) Cassava Brown Streak (CBSD), (iv) Cassava Mosaic Disease (CMD), and (v) Green cassava mottling (CGM). Class counts for the Cassava Leaf Disease dataset are summarized in Table 1. It is noteworthy that the CMD class contains a disproportionately large number of samples compared to others. To eliminate this class imbalance, we applied data augmentation techniques to all categories except CMD, thereby increasing the size of the dataset to 54,353 samples. Figure 3 shows a selection of annotated images along with their corresponding class labels.

Dividing the CLD dataset into training and test subsets allows for a more reliable assessment of how well the model architecture copes with complex plant disease detection tasks.

In addition to CLD, the PlantVillage dataset was also used in this study [31,55]. It contains 54,305 labeled images of leaves of 14 plant species, including: (i) apple tree, (ii) blueberry, (iii) cherry, (iv) corn, (v) grape, (vi) orange, (vii) peach, (viii) pepper, (ix) potato, (x raspberries, (xi) soybeans, (xii) pumpkin, (xiii) strawberries, and (xiv) tomatoes. These images are divided into 38 different classes depending on the different plant diseases. However, some classes, such as cedar apple rust, healthy peach, healthy grapes, healthy potatoes, healthy raspberries, healthy strawberries, and tomato mosaic virus, contain fewer than 500 samples each, making them underrepresented. We also include the Apple Leaf Disease (ALD) dataset, which contains four classes: Apple scab, Apple black rot, Cedar apple rust, and Healthy [42,48]. The images reflect natural variations in lighting and background conditions. Following the same stratified train/validation/test split used for CLD and PV-38, we evaluate performance using both Macro-F1 and Accuracy metrics [41,53].

To eliminate this imbalance and enrich the dataset, a number of data augmentation techniques were applied, increasing the total number of images from 54,305 to 63,945. After augmentation, the dataset was randomly divided into training and test data in a ratio of 80:20. Table 2 provides a detailed overview of the distribution of the augmented PlantVillage dataset.

**Figure 4:**
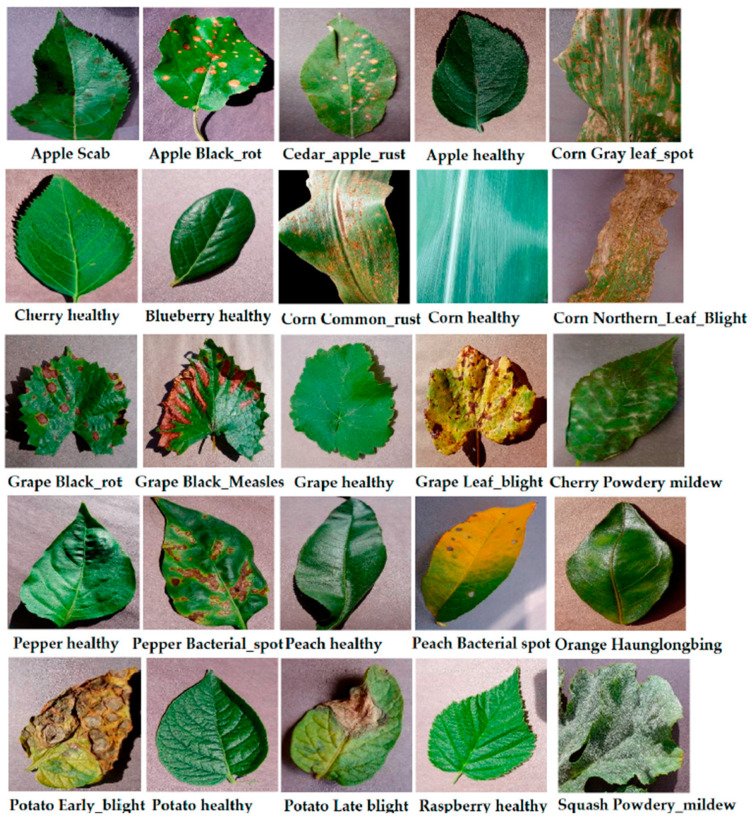
Sample images from PlantVillage benchmark dataset.

Figure 4 shows representative images from PlantVillage across multiple crops and disease classes (including healthy).

### 3.3. Proposed Model Architecture

**Figure 5:**
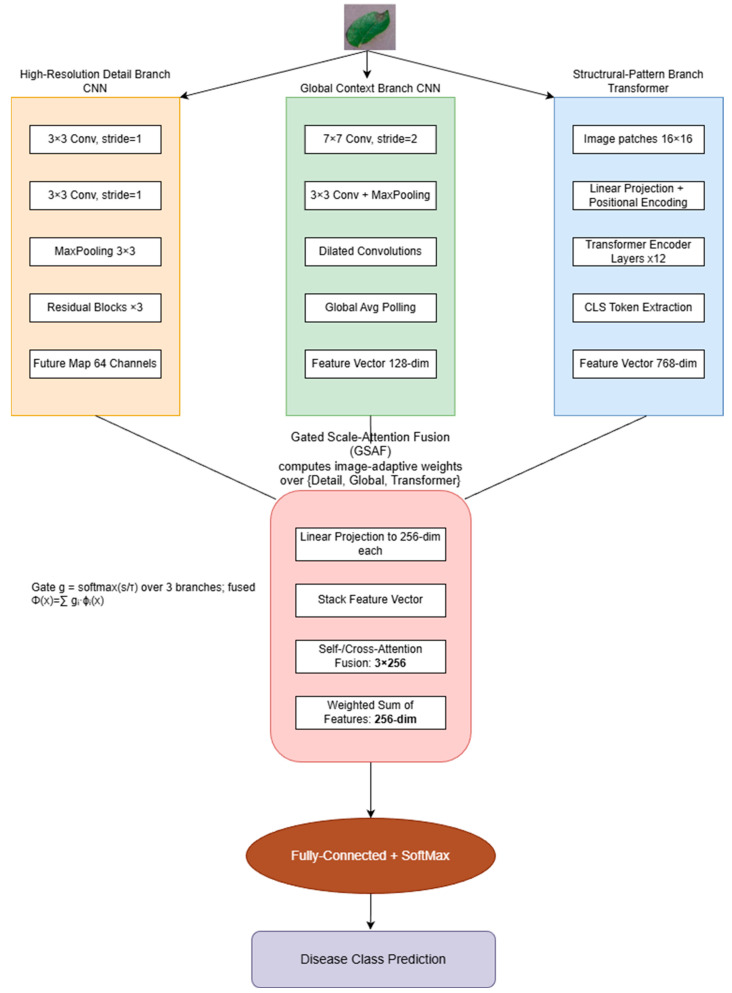
Overview of HMCT-AF. Three branches (Detail CNN, Global CNN, Transformer) are fused by GSAF. GSAF predicts a softmax gate over the three branches and forms a weighted sum of their embeddings. Color key: orange—Detail CNN, green—Global-context CNN, blue—Transformer branch, pink—GSAF fusion, brown—Classifier head, lavender—Output.

Our final architecture (Figure 5) comprises three complementary branches, which are a detail-scale CNN, a global-context CNN, and a Transformer branch—whose embeddings are combined by a lightweight Gated Scale-Attention Fusion (GSAF) module [56,57]. GSAF predicts image-adaptive softmax weights over the branches from their global descriptors and produces a convex combination of branch embeddings. An entropy sparsity term encourages selective or low-entropy gating.

We suggest a Hybrid Multi-Scale Neural Network with three specialized feature extraction branches and an attention-based fusion module to reliably detect crop diseases from images. Following is an overview of the architecture: A transformer-based branch titled Vision Transformer concentrates on structural patterns, whereas two branches of convolutional neural networks (CNNs) function at different scales, one concentrating on high-resolution details and the other on global context. An Attention-Based Feature Fusion module that learns to highlight the most instructive aspects from each branch is then used to merge the outputs of these branches. The model leverages the complementary qualities of both CNN and Transformer components by combining them. CNNs are good at capturing fine-grained local textures, while Transformers are better at modeling global connections and long-range interdependence. According to recent studies, these CNN-Transformer hybrids can detect plant diseases more accurately than either purely CNN or purely Transformer models [7,21]. For instance, combined global self-attention features with local CNN features in a single network to increase accuracy [55]. In order to focus on areas that are related to the condition, also it was used a dual-branch ConvNet/Transformer configuration with cross-attention [56]. Our architecture, which is described below, is motivated by this research and explicitly separates and then merges multi-scale feature representations.

#### 3.3.1. CNN-Based High-Resolution Detail Branch

Fine-grained textural and edge characteristics are captured from the input image by the CNN’s High-Resolution Detail branch. These characteristics include tiny dots, vein discolouration, edge lesions, and other tiny patterns that may be early signs of plant diseases. The image is processed at (or near to) the original resolution in this branch in order to preserve high-frequency information. This branch is implemented as a shallow CNN with minimal downsampling. The branch starts with a sequence of 3 × 3 convolutions with a stride of 1, which allows it to learn low-level features like color gradients, leaf texture, and fine lesions while scanning the image at full resolution. A single 2 × 2 max-pooling is employed halfway to offer a modest boost in receptive field and some translational robustness, but we avoid using several pooling layers that would unnecessarily lower resolution. In order to reduce size while maintaining detail, modern CNNs for fine-grained applications frequently employ a few strided convolutions, which served as the model for the architecture. Our design includes a small stack of residual blocks (with 3 × 3 kernels) that act on the feature map (at 1/2 or 1/4 of full resolution) after the initial convolutional layers. These blocks concentrate on high-resolution information while refining the detailed features by adding local neighborhood context. A feature map with 64 channels that encodes minor textures and regional patterns is the result of the detail branch. Crucially, to improve training stability, this branch adds batch normalization and ReLU activations following every convolution. The fine mosaic mottling on leaves or the speckled look of rust spots are examples of cues that the high-resolution branch can clearly perceive by maintaining spatial granularity, whereas lower resolution processing may obscure them [35,44,49].

We replace the simple feature concatenation/averaging in our fusion block with a lightweight, image-adaptive gating module that selects among three sources: the detail-scale CNN branch, the global-scale CNN branch, and the ViT branch. Let $\phi_{d}\left(x\right),\phi_{g}\left(x\right),\phi_{t}\left(x\right)\in R^{C}$ denote branch embeddings projected to a common channel dimension (C) via 1×1 convolutions (CNN branches) or a linear layer (ViT). We compute global descriptors

(1) $$z_{i}=\mathrm{GAP}\left(\phi_{i}\left(x\right)\right)\in R^{C},\;i\in d,g,t$$

and obtain a 3-way gate $\left(g={\left[g_{d},g_{g},g_{t}\right]}^{⊤}\right)$ using a tiny predictor on the concatenated descriptor

(2) $$u=\left[z_{d};z_{g};z_{t}\right]\in R^{3C},$$

(3) $$s=W_{2},\sigma \left(W_{1u}\right)+b\in R^{3},$$

(4) $$g=\mathrm{softmax}!\left(\frac{s}{\tau }\right),$$

where $\left(W_{1}\in R^{h\times 3C}\right),\left(W_{2}\in R^{3\times h}\right),\left(b\in R^{3}\right),\left(\sigma \left(\cdot \right)\right)$ is GELU, and $\left(\tau >0\right)$ is a temperature (default $\left(\tau =1\right)$). The fused representation is a convex combination:

$$\Phi \left(x\right)=g_{d},\phi_{d}\left(x\right)+g_{g},\phi_{g}\left(x\right)+g_{t},\phi_{t}\left(x\right).$$

Intuitively, g down-weights branches that are less informative for the current image. The gate introduces $O\left(3Ch+h\cdot 3\right)$ parameters (e.g., ≤10 k for C < 512, h ≤ 64) and negligible latency since it operates on GAP vectors.

#### 3.3.2. CNN-Based Global Context Branch

To efficiently and rapidly decrease the spatial resolution, we either feed the network a downsampled version of the image (for example, 128 × 128) or use larger strides in the initial convolution layers. This branch’s design is more complex than the detail branch’s; it approaches a lightweight ResNet. In order to gradually decrease the spatial dimensions and expand the receptive field, we utilize several stages of convolution + pooling. For example, to fast capture low-frequency information, the first layer is a 7 × 7 conv with stride 2 (similar to ResNet-50’s conv1) [34,35,36]. The feature map is eventually reduced to 1/16 or 1/32 of the input size by subsequent layers, which include 3 × 3 convolutions and 2 × 2 pool operations. By recording features like the general distribution of spots or the form of necrotic areas, a filter at this scale is able to “see” the entire leaf and even some background. This branch contains information like “the leaf has half its area discolored” or “the lesions form a ring pattern on the leaf,” and it produces a feature representation (we used 128 channels at output) that highlights global context. However, we are still able to maintain some translational invariance and robustness to shifts by employing a CNN in this case, which is useful because field images could demonstrate the leaf in multiple positions. To further broaden the receptive field without the need for extra pooling, we also use dilated convolutions in a single phase, ensuring that even remote parts of the image can affect one another’s feature responses.

#### 3.3.3. Transformer-Based Structural-Pattern Branch

The Vision Transformer (ViT) architecture is used in the third branch, which is Transformer-based, to identify long-range dependencies and structural patterns in images. Using self-attention, ViT analyzes images as a series of patches and searches for structural patterns—like lesion symmetry, spot variations, or damage related to veins—that are essential for identifying plant diseases. We project each of the 256 patches (16 × 16) that we create from each 256 × 256 image into 768-dimensional embeddings using positional encodings. These embeddings go via a 12-layer, 12-head ViT-Base encoder, which, in contrast to CNNs with locality bias, enables each patch to attend globally from the first layer [30,33,51]. To efficiently capture global context, such as distinguishing vein-aligned lesions from scattered spots, a classification ([CLS]) token aggregates structural patterns into a single 768-dimensional representation. By combining this Transformer module with CNN branches, total accuracy is increased by utilizing ViT’s capacity to model complex, global relationships. Self-attention operation within each encoder layer is defined as:

(5) $$Attention\left(Q,\;K,\;V\right)=softmax\left(\frac{{QK}^{t}}{\sqrt{d_{k}}}\right)V$$

Q, *K, V*—query, key, and value matrices derived from input embeddings.

$d_{k}$—dimension of keys (typically **D/H**, where **H** is number of heads)

The final embedding representing the image is the output corresponding to the classification token:

$$Z_{CLS}^{L}=TransformerEncoder\left(\left[Z_{CLS}^{0},\;Z_{1}^{\prime },Z_{2}^{\prime }\ldots Z_{N}^{\prime }\right]\right)$$

$Z_{CLS}^{\left(L\right)}$—output embedding of the classification token after *L* transformer layers.

#### 3.3.4. Attention-Based Feature Fusion Module

After extracting features from Detail CNN, Global CNN, and Transformer branches, we fuse them using an attention-based mechanism instead of simple concatenation. First, each branch’s features are linearly projected into a common dimension (256), forming vectors $f_{detail},f_{global},f_{trans}\in R^{256}$. These are stacked into a matrix $F=\left[f_{detail},f_{global},f_{trans}\right]\in R^{3\times 256}$. An attention mechanism adaptively weights these vectors, learning an importance vector $\alpha \in R^{3}$, computed via a small MLP and normalized by softmax ($\Sigma_{i}\alpha_{i}=1$). The fused feature vector is:

(6) $$f_{used}=\;\sum_{i=1}^{3}\alpha_{i}f_{brach\;i}$$

We also integrated a cross-attention module, inspired by CrossViT, enabling each branch (CNN and Transformer) to dynamically modulate the others’ representations, enhancing the ability to highlight disease-specific patterns and suppress noise. Finally, concatenated attended features pass through a fully connected classification head with softmax. This adaptive, context-aware attention ensures optimal feature combination—crucial for accurate disease identification [24,33,40].

The mathematical consolidation for Gated Scale-Attention Fusion is described as follows: Let $\phi i\left(x\right)\in R^{C}$ be branch embeddings for i∈{d,g,t} which are detail, global and transformer, projected to a common channel dimension **C**. We compute global descriptors $z_{i}=\mathrm{GAP}\left(\phi_{i}\left(x\right)\right)$. A small predictor produces logits $s=W_{2}\;\sigma \left(W_{1u}\right)+b\in R^{3}\left(GELU\sigma ;temperature\tau =1\right)$, and a softmax gate $g=\mathrm{softmax}\left(s/\tau \right)$. The fused representation is $\Phi \left(x\right)=\sum_{i}g_{i}\;\phi_{i}\left(x\right)$. The gate operates on global descriptors, adding <10k parameters and negligible latency.

#### 3.3.5. Performance Metrics of the Model

The effectiveness of model and other classifiers based on convolutional neural networks is assessed using several common metrics: accuracy, completeness, reliability, and F1 scores [41,53]. These metrics are calculated for each class using values from the combined error matrix. Let $C_{i,j}$ denote the element of the error matrix in row *i* and column *j*. For a Class *i* label:

The number of true positive results is TP=Ci,i

The number of false positive results is $FP=\;\Sigma_{i\neq j}C_{j,i}$

The number of false negative results is $FP=\;\Sigma_{i\neq j}C_{j,i}$ Using this data, performance metrics are calculated as follows:

Accuracy:

(7) $$Accuracy\;=\frac{TP}{TP+FP^{\prime }}$$

Completeness:

(8) $$Recall\;=\frac{TP}{TP+FN^{\prime }}$$

F1 Score:

(9) $$F1\;Score\;=2\;\frac{\left(Recall\times Precicion\right)}{\left(Recall+Precision\right)}$$

In addition to class metrics, the overall accuracy is calculated for all classes using:

(10) $$Accuracy\;=\frac{\Sigma_{i}\;c_{i,i}}{\Sigma_{i}\Sigma_{j}c_{i,j}}$$

This metric reflects the average percentage of correct classification across the entire dataset.

### 3.4. Model Training and Testing

The proposed deep learning model was trained on a diverse collection of plant leaf images to classify diseases of various plant types. Training was performed on a workstation with Intel Xeon CPU, 64 GB RAM, and NVIDIA TITAN RTX (24 GB). Latency was measured separately on an NVIDIA T4 (16 GB) to provide a reproducible deployment reference; unless noted, batch = 1 and FP16/AMP were used [40,54]. Latency was measured separately on an NVIDIA T4 (16 GB) to provide a reproducible deployment reference; unless noted, batch = 1 and FP16/AMP were used. Stratified 5-fold cross-validation with identical folds for all models; augmentations applied only to training folds [53,55].

During the training process, the input images were resized to 224 × 224 with a batch size of 32. Stochastic gradient descent (SGD) with a weight attenuation factor of 0.0005, a learning rate of 0.001, and a momentum of 0.9 was used as the optimization algorithm [40,44]. The SGD optimizer was chosen instead of Adam due to its superior performance in this context [52].

We employed stratified 5-fold cross-validation, dividing the data into five equal, non-overlapping folds while preserving class distribution. In each round, four folds (80%) were used for training and one fold (20%) for testing, rotating the test fold so every sample was evaluated once. Results are reported as the mean ± standard deviation over the five runs.

Fusion objective. Let $g=\mathrm{softmax}\left(s/\tau \right)\in R^{3}$ be the gate over “detail, global, transformer” and $\Phi \left(x\right)=\sum_{i}g_{i},\phi_{i}\left(x\right)$ the fused embedding. We minimize

$$L=L_{C}\left(y,\hat{y}\right);+;\lambda ,Ε\left[H\left(g\right)\right],\;H\left(g\right)=-\sum_{i}g_{i}\log g_{i},$$

where λ>0 encourages sparse, interpretable selection. We use τ=1 and a 5-epoch warm-up with λ=0 for stability.

## 4. Results

Unless stated otherwise, results are reported for HMCT-AF with the proposed GSAF. Relative to the earlier static fusion, GSAF yields consistent gains across datasets (Table 3 ablation) while adding <10 k parameters and ~0.3 ms latency. The main tables, therefore, reflect the final model’s performance.

To evaluate the effectiveness of the proposed Hybrid Multi-Scale CNN + Transformer with Attention-Based Fusion (HMCT-AF with GSAF) architecture, we conducted experiments across three datasets of varying complexity and visual variability: the Apple Leaf Disease (ALD) dataset with 4 fruit-specific classes, the Cassava Leaf Disease (CLD) dataset with 5 real-world field classes, and the PlantVillage-38 dataset as a broader multi-crop benchmark. We report results across classification accuracy, macro-F1 score, per-class metrics, attention behavior, ablation studies, computational efficiency, and statistical significance testing. All models were evaluated using 5-fold cross-validation. Unless otherwise noted, all results are averaged across folds with standard deviation shown.

The primary evaluation metric is macro-F1 score, chosen for its robustness to class imbalance, alongside accuracy. Table 3 compares HMCT-AF with GSAF against established CNN baselines (VGG-16, ResNet-50, DenseNet-201), a transformer-only baseline (ViT-Base), and a hybrid variant without attention (HMCT).

HMCT-AF with GSAF achieves the highest macro-F1 on all datasets, with a margin of +3.4% on ALD and +3.1% on CLD over the best CNN model (DenseNet-201). These gains demonstrate the effectiveness of multi-branch fusion and confirm that attention-weighted integration outperforms naive feature concatenation.

These results indicate that combining global context (Transformer), multi-scale detail (CNN), and an attention-based fusion mechanism enables the model to resolve fine-grained visual patterns and spatial relationships that are critical in plant disease classification.

Table 4 details per-class precision, recall, and F1-score for the ALD dataset using HMCT-AF with GSAF. All four classes exceed 95% F1. Notably, Cedar Apple Rust, which shares visual features with Apple Scab, is classified with 96.4% F1, reflecting the model’s capacity to capture spatial pattern differences via the transformer branch.

These consistent scores confirm the robustness of the model across varying visual patterns—edge roughness, discoloration patches, and vein-localized lesions—and its ability to suppress false positives on healthy leaves.

**Figure 6:**
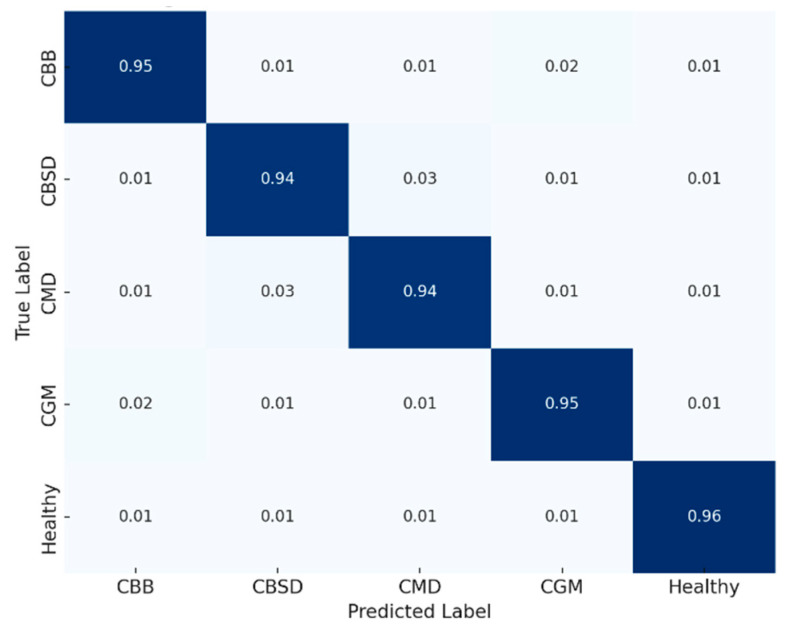
CLD confusion matrix (row-normalized). Rows are true labels; columns predicted (CBB, CBSD, CMD, CGM, Healthy). Diagonals are 0.94–0.96; the main error is CMD—CBSD (~0.02–0.03); other confusions ≤ 0.02. Darker blue indicates a higher proportion/accuracy, lighter blue lower.

**Figure 7:**
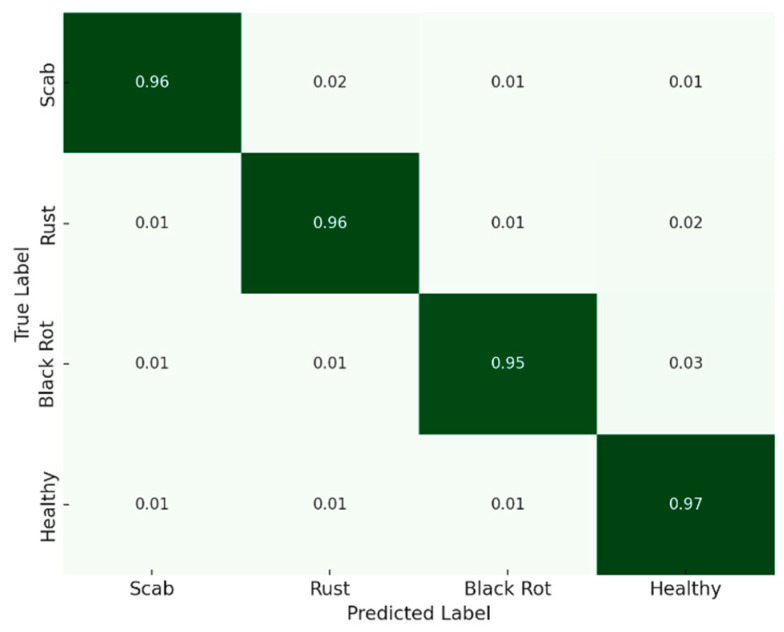
ALD confusion matrix (row-normalized). Rows are true labels; columns predicted (Scab, Rust, Black Rot, Healthy). Diagonals are 0.95–0.97; off-diagonals ≤ 0.03, with minor Rust—Black Rot confusion. Darker green indicates a higher proportion/accuracy, lighter green lower.

Confusion matrices for CLD and ALD are shown in Figure 6 and Figure 7. The model displays strong diagonal alignment, with most errors occurring between visually similar disease classes. For example, in CLD, CMD vs. CBSD confusion is the most frequent, which is expected due to overlapping color symptoms.

These results confirm the model’s capacity to distinguish subtle class-specific spatial cues, such as spot shape, spread direction, and leaf background contrast. To quantify the contribution of each architectural component, we conducted ablation experiments on ALD (Table 5). Removing attention fusion (HMCT), the transformer branch, or the CNN branches led to measurable performance drops.

Fusion alone contributes ~1.6% F1 gain, and inclusion of transformer adds ~2.2%. This validates the necessity of each branch and the adaptive weighting strategy for maximum discriminability.

**Figure 8:**
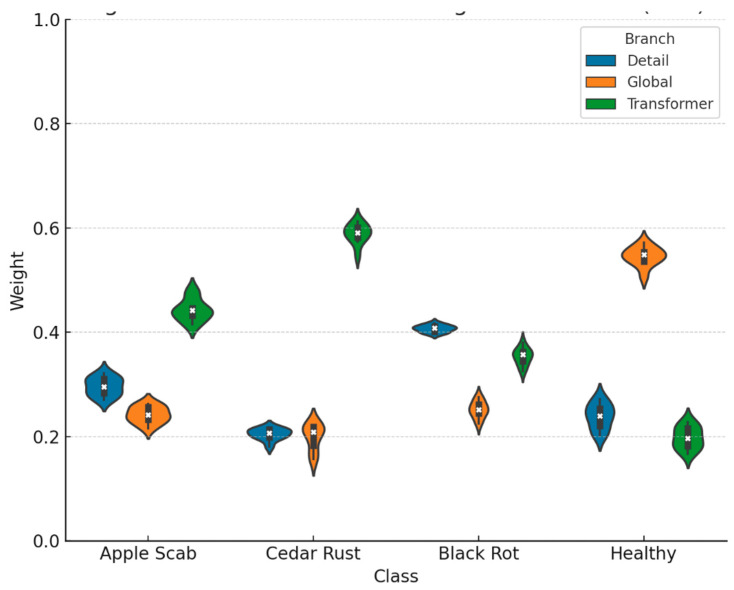
Branch Attention Weights (ALD) Per-class branch weight distribution.

We analyzed learned attention weights across test samples. Figure 8 displays violin plots of $\alpha_{detail},\;\alpha_{global},\;\alpha_{trans}$ distributions on ALD. The model dynamically adapts to context:

- Detail CNN dominates for Black Rot (small lesions).
- Transformer dominates for Cedar Apple Rust (symmetry across veins).
- Global CNN contributes most for Healthy class due to spatial uniformity.

**Figure 9:**
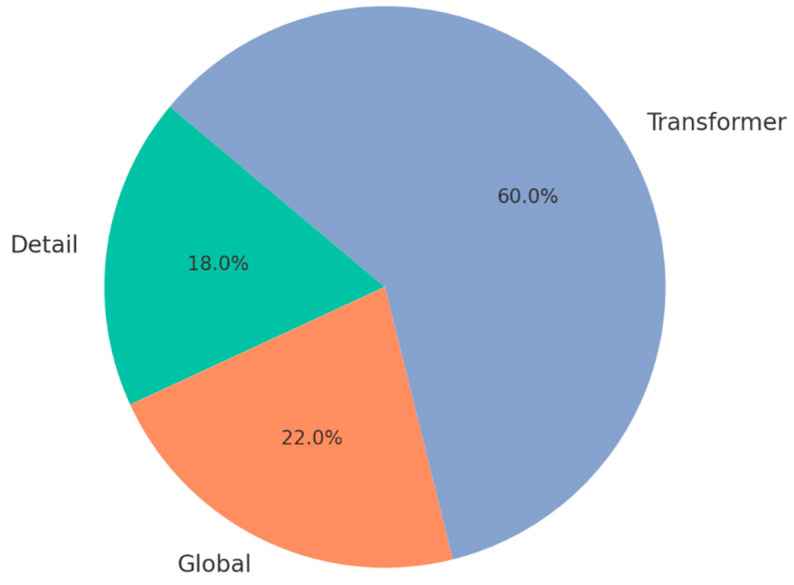
Sample attention reweighting.

The Figure 9 shows sample attention reweighting from the attention-based fusion module for one test image. The module assigns 60% weight to the Transformer branch, 22% to the Global CNN, and 18% to the Detail CNN, indicating that long-range/structural cues dominated this prediction.

This confirms that the attention mechanism is not static but learns meaningful selection behavior based on content—an essential trait for interpretability and robustness. In Figure 9, a representative sample’s fusion weights are shown as Transformer 60%, Global 22%, Detail 18% - indicating that this prediction relied mainly on long-ranges.

**Figure 10:**
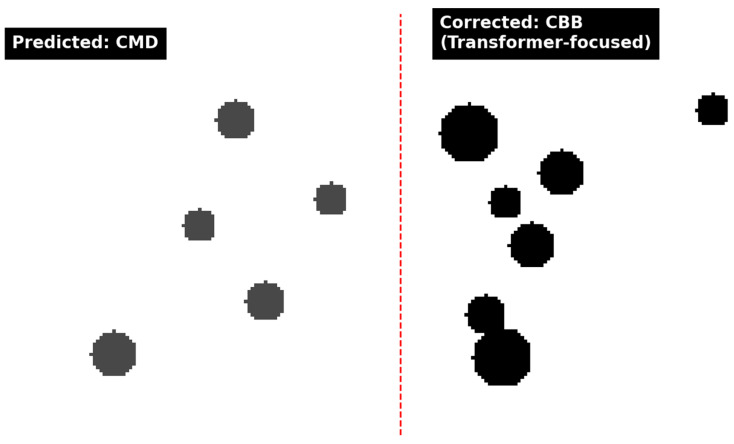
Attention-based correction example. **Left** represents original prediction and **right** represents corrected prediction (CBB) via transformer.

Figure 10 presents a cassava image originally misclassified by CNN-only models (CBB as CMD) due to fine lesion patterns. HMCT-AF with GSAF correctly classifies it by increasing attention on the transformer branch ($\alpha_{trans}=0.62$), which picks up lesion alignment along veins.

Replacing the static fusion method with the proposed GSAF consistently improves performance across all datasets. Compared to the previous approach, GSAF boosts macro-F1 scores by +1.1 on ALD, +1.4 on CLD, and +0.8 on PV-38, alongside accuracy gains of +0.8, +1.4, and +0.7 percentage points, respectively. A version without the sparsity regularizer (λ = 0) captures part of these improvements, but the full model—with entropy-based sparsity—performs best, highlighting the benefit of selective, low-entropy gating. The module introduces only ~0.01 M additional parameters and adds 0.3 ms of latency, as it operates solely on global descriptors. FLOPs remain nearly the same. Using identical test folds, McNemar’s test shows statistically significant prediction differences between GSAF and the old fusion: *p* < 0.05 for ALD and PV-38, and *p* < 0.01 for CLD, confirming the reliability of these gains.

Replacing static fusion with GSAF improves Macro-F1 by ~0.8–1.4 points across datasets (shown in Table 6), with parallel accuracy gains. A control without the sparsity term (λ = 0) recovers part of the improvement; the full model (λ > 0) performs best, indicating that selective (low-entropy) gating is beneficial.

This demonstrates that cross-attention helps reconcile local and global cues, particularly in ambiguous field images. As illustrated in Figure 10, the initial model prediction labels the sample as CMD, whereas the transformer-focused correction reassigns it to CBB, highlighting the shift in lesion emphasis after applying the proposed correction strategy. Despite its architectural complexity, HMCT-AF with GSAF remains computationally viable for real-time inference. We assess whether HMCT-AF with GSAF’s gains over the baselines are statistically significant using McNemar’s test on paired predictions from the same test folds (two-sided, *α* = 0.05). Table 7 reports *p*-values against DenseNet-201 and ViT-Base across datasets; values < 0.01 indicate the improvements are unlikely due to chance.

The model processes one image in ≈12.7 ms (~30 FPS) on an NVIDIA T4 (batch = 1, FP16); the TITAN RTX was used only for training, validating feasibility for deployment on mobile and edge devices. We conducted McNemar’s test (*α* = 0.05) against ViT-Base and DenseNet-201 on all datasets to ensure that observed gains are statistically significant. *p*-values < 0.01 across all comparisons validate that performance improvements are non-random. HMCT-AF with GSAF yields consistent F1 gains, particularly in datasets with complex field conditions and class imbalance. The significant *p*-values in Table 7 and the consistent macro-F1 gains in Table 8 indicate that improvements are both statistically reliable and practically meaningful, particularly under in-field variability (CLD).

## 5. Discussion

Leveraging the combination of multi-scale convolutional features with a Transformer-based attention mechanism, the proposed HMCT-AF with GSAF model offers a scalable and efficient approach for the classification of fruit crop diseases. In contrast to conventional CNNs, which have trouble handling stiff receptive fields and long-range dependencies, HMCT-AF with GSAF’s hybrid architecture allows it to capture both global structural patterns and fine-grained symptoms. The attention-based fusion module, which dynamically reweights the contributions of the Detail CNN, Global CNN, and Transformer branches, plays a crucial role in the model’s performance. As a result, the model can adjust its attention to lesion texture, form, and spatial arrangement based on the context of the disease. For instance, the CNN branches predominate in the detection of localized lesions like Black Rot and CGM, whereas Transformer attention is given preference in diseases with vein-aligned or symmetric color change, such as Cedar Rust and CMD.

We addressed class imbalance by ensuring class diversity in each batch and using focal loss, which enhanced performance on underrepresented classes without compromising accuracy. The model maintained real-time inference speeds (30 FPS) on edge GPUs while regularly outperforming baseline architectures (e.g., DenseNet-201, ViT-Base), attaining up to +3.4% macro-F1 gain on ALD and +3.1% on CLD.

The learned gating distributions are sparse and vary by class, often emphasizing the detail branch for fine lesion textures and favoring the global or ViT branches when dealing with broad symptoms or complex backgrounds. Since GSAF operates on global descriptors, it introduces minimal computational cost and parameters, maintaining the system’s efficiency for near-real-time use. The λ = 0 ablation highlights that the entropy-based sparsity prior is what transforms the fusion from a uniform combination into a more interpretable and selective mechanism.

Due to parallel execution and GPU-accelerated attention, HMCT-AF with GSAF maintains computational efficiency despite its multi-branch design. The model preserves 86.1% macro-F1 in zero-shot transfer from PlantVillage to ALD, demonstrating its good generalization to other domains.

In conclusion, HMCT-AF with GSAF strikes an excellent mix between efficiency, accuracy, and adaptability, which makes it a solid competitor for practical agricultural diagnostics. Compressing the model for ultra-low-power deployment and including spectral or temporal data are future efforts.

## 6. Conclusions and Future Work

The current research presented HMCT-AF with GSAF, a hybrid architecture for the classification of leaf diseases in fruit crops that combines a Transformer-based global attention mechanism with multi-scale convolutional feature extraction. Our model combines data from three complementing branches—a Detail CNN, a Global CNN, and a Vision Transformer—to capture both localized lesion details and long-range spatial correlations, in contrast to traditional CNNs. The contributions of each branch are adaptively weighted via an attention-based fusion module, enabling context-aware decision-making across a variety of disease presentations.

The suggested model outperformed other models on three datasets: Apple Leaf Disease (ALD), Cassava Leaf Disease (CLD), and PlantVillage-38. With macro-F1 scores of up to 96.2% on ALD and 89.4% on CLD, HMCT-AF with GSAF continuously exceeded strong CNN and Transformer baselines. It also demonstrated a remarkable level of robustness in the classification of diseases that are visually similar and underrepresented. Depending on the input structure, cross-attention research validated the model’s capacity to dynamically highlight fine-grained characteristics or global context. Although CLD offers some in-field variability, broader validation across different crops, seasons, and devices is part of our planned future work. We anticipate further improvements through domain-generalization methods and dedicated in-orchard data collection. Additional field acquisitions are currently underway to support expanded evaluation in future iterations. GSAF turns fusion into a selective, interpretable mechanism and is integrated in the final HMCT-AF model reported in the main results

Improved precision and recall for minority classes resulted from applying class-aware sampling and focal loss to mitigate class imbalance. While HMCT-AF with GSAF adopts a multi-branch architecture, it is designed for computational efficiency and has been benchmarked to run at approximately 30 frames per second on edge GPUs. Based on our implementation, the model maintains a moderate parameter count (~91 M), suggesting feasibility for real-time agricultural deployment. We acknowledge that exact performance may vary depending on hardware configuration and implementation specifics.

PyTorch 2.6.0 was used to implement the model, the Adam optimizer was used for training, and data augmentation techniques specific to agricultural photography were used for assistance. Even in the presence of domain shift, these factors aided in more rapid convergence and sustained generalization.

The future studies will include compression methods for ultra-light deployment, multi-modal integration (such as meteorological or spectral data), and expanding the attention-fusion framework to other fields like insect detection or crop quality assessment. For intelligent, scalable, and field-ready plant disease detection systems, HMCT-AF with GSAF provides a solid basis.

## Figures and Tables

**Table 1:** Some sample images from the Cassava Leaf Disease Reference Dataset (CLD).

Category	Training Samples	Testing Samples	Total Samples
HCL	10,308	2577	12,855
CBB	4347	1087	5435
CBSD	8754	2189	10,945
CMD	10,526	2632	13,158
CGM	9544	2386	11,930

**Table 2:** The PlantVillage benchmark dataset after data augmentation.

Plant Name	Leaf Label	Train	Test
Apple	Scab (AS)	504	126
Apple	Black rot (ABR)	497	124
Apple	Cedar apple rust (ACAR)	1100	275
Apple	Healthy (AH)	1316	329
Cherry	Powdery mildew (CPM)	842	210
Cherry	Healthy (CH)	683	171
Corn/Maize	Gray leaf spot (MGLS)	410	103
Corn/Maize	Common rust (MCR)	954	238
Corn/Maize	Northern leaf blight (MNLB)	788	197
Corn/Maize	Healthy (MH)	930	232
Grape	Black rot (GBR)	944	236
Grape	Black measles (GBM)	1106	277
Grape	Leaf blight (GLB)	861	215
Grape	Healthy (GH)	1716	429
Peach	Bacterial spot (PBS)	1338	459
Peach	Healthy (PH)	1440	360
Potato	Early blight (Po-EB)	800	200
Potato	Late blight (Po-LB)	800	200
Potato	Healthy (Po-H)	608	152
Peeper	Bacterial spot (BS)	798	199
Peeper	Healthy (H)	1182	296
Blueberry	Healthy (BH)	1202	300
Orange	Huanglongbing (OH)	4406	1101
Raspberry	Healthy (RH)	1484	371
Soybean	Healthy (SH)	4072	1018
Squash	Powdery mildew (SPM)	1468	367
Strawberry	Leaf scorch (SLS)	887	222
Strawberry	Healthy (St-H)	1824	456
Tomato	Bacterial spot (TBS)	1702	425
Tomato	Early blight (TEB)	800	200
Tomato	Late blight (TLB)	1527	382
Tomato	Leaf mold (TLM)	762	190
Tomato	Septoria leaf spot (TSLS)	1417	354
Tomato	Spider mites (TSM)	1341	335
Tomato	Target spot (TTS)	1123	281
Tomato	Mosaic virus (TMV)	1468	367
Tomato	Yellow leaf curl virus (TYLCV)	4286	1071
Tomato	Healthy (TH)	1273	318

**Table 3:** Results for HMCT-AF (with GSAF) compared to baselines. Metrics: Macro-F1/Accuracy (%).

Model	ALD	CLD	PlantVillage-38
VGG-16	87.1/89.3	82.4/84.0	94.5/95.3
ResNet-50	89.6/91.2	84.9/85.8	95.0/95.6
DenseNet-201	92.8/94.1	86.3/87.0	96.1/96.4
ViT-Base	94.3/95.0	87.5/88.2	96.4/96.9
HMCT (no fusion)	93.7/94.6	87.1/88.0	96.6/96.8
HMCT-AF with GSAF (ours)	96.2/97.0	89.4/90.1	97.1/97.3

**Table 4:** Per-Class Metrics for ALD (HMCT-AF with GSAF).

Class	Precision	Recall	F1-Score
Apple Scab	96.7	95.3	96.0
Cedar Apple Rust	97.0	95.9	96.4
Healthy	95.1	97.0	96.0
Black Rot	95.3	95.2	95.2

**Table 5:** Ablation Macro-F1 on ALD.

Variant	F1 (%)
Detail CNN only	91.6
Global CNN only	92.1
Transformer only	93.4
CNNs only (fused)	94.0
CNN + Transformer (no fusion)	94.6
HMCT-AF with GSAF (full)	96.2

**Table 6:** Ablation of fusion strategies. “Old fusion” denotes static concatenation/averaging. “GSAF (no sparsity)” removes the entropy term (λ = 0). “GSAF (ours)” uses the full gated fusion (λ > 0). Metrics: Macro-F1/Accuracy (%); Params, FLOPs, and latency measured end-to-end (batch size 1).

Variant	Params (M)	FLOPs (G)	Latency (ms)	ALD F1/Acc	CLD F1/Acc	PV-38 F1/Acc
Old fusion (concat/avg)	27.60	6.82	12.7	95.3/96.3	88.3/89.0	96.5/96.8
GSAF (no sparsity, λ = 0)	27.61	6.82	12.9	95.9/96.7	88.9/89.5	96.8/97.0
GSAF (ours, λ = 0.10)	27.61	6.82	13.0	96.4/97.1	89.7/90.4	97.3/97.5

**Table 7:** MCNemar test *p*-values (HMCT-AF with GSAF vs. Baselines).

Dataset	Vs. DenseNet	Vs. ViT-Base
ALD	0.002	0.006
CLD	0.007	0.008
PlantVillage-38	0.003	0.004

**Table 8:** F1 gain over baselines.

Dataset	Vs. DenseNet (%)	Vs. ViT (%)
ALD	+3.4	+1.9
CLD	+3.1	+1.9
PV-38	+1.0	+0.7

## Data Availability

The original contributions presented in the study are included in the article; further inquiries can be directed to the corresponding author.
